# Pacing Profiles in Half-Marathon and Marathon Running Based on Performance Level, Sex, and Age: A Narrative Review

**DOI:** 10.3390/medicina62081503

**Published:** 2026-08-05

**Authors:** Ljubica Ristanović, Stanimir Stojiljković, Ivan Ćuk, Katja Weiss, Thomas Rosemann, Beat Knechtle

**Affiliations:** 1 Faculty of Sport and Physical Education, University of Belgrade, 11030 Belgrade, Serbia; ljubicapapic20@gmail.com (L.R.); stanimirstojiljkovic@gmail.com (S.S.); ivan_cuk84@yahoo.com (I.Ć.); 2 Institute of Primary Care, University of Zurich, 8091 Zurich, Switzerland; katja@weiss.co.com (K.W.); thomas.rosemann@usz.ch (T.R.); 3 Medbase St. Gallen Am Vadianplatz, 9000 St. Gallen, Switzerland

**Keywords:** pacing strategy, marathon, half-marathon, long-distance running, pacing variability, performance level, sex differences, fatigue

## Abstract

*Background and Objectives*: Pacing behavior has been consistently associated with performance in long-distance running, yet its patterns across different race distances and population subgroups remain insufficiently understood. Therefore, this narrative review synthesizes and critically discusses available evidence on pacing in half-marathon and marathon running, considering both within- and between-race differences, with a particular focus on performance level, sex, and age. *Materials and Methods*: A structured, non-systematic search was conducted in PubMed, SPORTDiscus, Scopus, and Web of Science, with Google Scholar used as a supplementary source. Peer-reviewed English-language full-text studies reporting pacing-related outcomes were included, and findings from 37 studies published up to March 2026 were synthesized narratively. *Results*: Available observational evidence suggests differences in pacing profiles between race distances, with generally more even pacing in the half-marathon and more positive pacing in the marathon. Performance level showed the strongest association with pacing behavior: faster runners exhibited more stable pacing patterns, whereas slower runners showed greater variability and a more pronounced pace decline. Sex differences appeared more evident in marathon running, where women generally demonstrated more even pacing than men, whereas age-related differences were small and inconsistent. Comparisons between race distances suggest that pacing variability may increase with longer race duration and lower performance level. *Conclusions*: Overall, performance level showed the most consistent relationship with pacing behavior in long-distance running. In mass-participation events, observed pacing profiles may reflect fatigue-related changes and inaccurate pace selection rather than a deliberate strategy. More even or slightly negative pacing has generally been associated with more favorable performance outcomes, but this should be interpreted as an observed association rather than a direct prescriptive recommendation. The proposed practical reference points should be viewed as tentative, evidence-informed aids for interpreting pacing behavior according to performance level and race distance, while using sex and age as secondary contextual factors.

## 1. Introduction

Long-distance running has expanded substantially worldwide, particularly among recreational runners, older age groups, and women [1,2,3,4,5,6,7]. This broader participation has coincided with slower average finishing times in some mass-participation events [3,8], while elite performances have continued to improve [2]. These parallel trends suggest that long-distance running performance should be interpreted in relation to participation structure, performance level, and training- and technology-related developments [9,10].

Race duration differs markedly between elite and recreational runners. Elite half-marathon and marathon performances are completed in under ~1 h and ~2 h, respectively, whereas slower recreational runners may require more than 3 h and 6 h [11,12,13]. Longer race duration may increase physiological strain and prolong disturbances to homeostasis [14,15], while marathon racing may also induce transient systemic stress and musculoskeletal injury despite the broader health benefits of endurance training [16]. Because recreational runners represent most participants and often run for health-related reasons [17], approaches that limit excessive fatigue while supporting endurance performance are of particular practical importance.

Pacing strategy has consistently been associated with endurance performance [18,19,20,21,22,23,24,25,26,27,28] and varies according to race distance [29,30,31], performance level, sex, and age [27,32,33,34]. It may also change over time and across competitive contexts; for example, elite marathon pacing over the past 50 years has shifted from pronounced positive pacing toward more even or negative profiles [35]. However, large-scale road-race analyses are constrained by split-time availability, commonly recorded every 5 km, which limits pacing resolution, particularly in shorter events. Consequently, research has focused predominantly on marathons [27,36,37,38,39,40,41], with fewer studies examining the half-marathon [42,43], despite its high participation rates [44], whereas shorter distances, such as 10 km, are more often examined in controlled experimental settings [45].

Beyond race-distance differences, a recent systematic review [46] suggests that pacing appears to play a more prominent role as race distance increases, alongside other factors such as training and environmental conditions. However, the available review literature has focused predominantly on marathon pacing and has not comprehensively integrated evidence comparing half-marathon and marathon races while simultaneously considering performance level, sex, and age [40]. Therefore, the novelty of this narrative review lies in synthesizing pacing evidence across these two race distances and key population subgroups within a single interpretative framework, while explicitly considering differences in the available evidence base between race distances. This approach provides a broader perspective on pacing behavior across different research groups, race settings, and athlete populations.

### 1.1. The Concept and Significance of Pacing Strategy in Long-Distance Running

Some definitions indicate that pacing strategy encompasses the distribution of energy reserves, strength, and speed throughout the entire race without significant deceleration [47,48], or, in other words, the distribution of effort intensity during a race. It has been described as a dynamic process integrating prior experience, knowledge of the race endpoint, and sensory feedback [22,28,49].

In this review, pacing strategy refers to the planned regulation of effort during a race, whereas pacing behavior describes the observed changes in running speed. The term pacing profile refers to the overall distribution of speed throughout a race, while pacing pattern describes the specific characteristics or shape of that distribution.

Contemporary neurophysiological and psychophysiological models conceptualize pacing as an anticipatory and feedback-driven process in which central regulation, afferent physiological feedback, perceived exertion, prior experience, knowledge of the remaining distance, and the desired competitive outcome are integrated to regulate exercise intensity [22,49,50,51,52]. More recent psychophysiological evidence further suggests that pacing behavior during prolonged endurance exercise reflects nonlinear interactions between sensory-discriminatory, affective-motivational, and cognitive-evaluative dimensions, which help align planned behavior with the current physiological state and competition context [52]. Within this framework, athletes do not simply respond passively to physiological disturbance but adjust effort according to the expected duration of the task, the perceived rate of homeostatic disturbance, and the anticipated ability to tolerate effort until the endpoint [22,48,50,51]. Accordingly, a pacing strategy may be planned before the race, whereas pacing behavior is continuously modified throughout the race in response to internal and external demands [49]. Premature exhaustion may occur if the selected pace is too high at any point during the race [49].

External factors such as environmental conditions and course characteristics have been shown to influence performance and, consequently, pacing behavior [53,54,55,56,57,58,59,60], while internal factors include glycogen availability, thermoregulation, neuromuscular fatigue, and perceived exertion [22]. Recent evidence suggests that energy availability, particularly carbohydrate status, influences pacing behavior not only through metabolic constraints but also via perceptual and cognitive mechanisms, affecting effort regulation even before physiological limits are reached [61].

In this context, the level of effort during running, commonly expressed as a percentage of maximum oxygen uptake (%VO_2_max), is closely associated with pacing regulation. Concepts such as critical speed [62] and critical power [63] provide a useful theoretical framework for interpreting the boundary between sustainable and unsustainable exercise intensity [64,65]. However, most studies included in this review inferred pacing behavior from split times and segmental speeds rather than from direct physiological measurements. Therefore, running pace, particularly on relatively flat courses, should be interpreted as a practical proxy for effort rather than as a direct physiological measure [66].

Maintaining an appropriate pacing strategy has been associated with limiting excessive physiological strain and sustaining performance [22,48,67,68]. It may also have practical relevance for improving race experience in recreational runners [69], although evidence directly linking pacing behavior to injury risk remains limited [68].

### 1.2. Pacing Profiles in Long-Distance Running

The resulting pacing behavior may be associated with race duration, fatigue-related changes in power output [48,50,70], experience [71,72], and the athlete’s physiological capacity [49,73].

Common pacing profiles include even, positive (progressive slowing), and negative (progressive speeding) pacing, whereas variable pacing refers more broadly to non-uniform speed changes that do not follow a consistently even, positive, or negative profile [68,74]. Some profiles may exhibit more complex non-monotonic patterns, such as reverse J-shaped (fast start followed by gradual slowing and an end spurt) [70,73,75,76] and U-shaped distributions [70,77]. U-shaped patterns have been reported, particularly in shorter, tactically driven events, where early positioning and finishing speed are important [70,77]. In contrast, such patterns are uncommon in longer endurance events, where pacing may be more strongly associated with physiological and energetic factors [46].

Pacing variability is commonly operationalized using quantitative indicators such as absolute change in speed (ACS), coefficient of variation (CV), and pace range, which provide useful indicators of within-race speed fluctuations [78]. ACS reflects the average magnitude of deviation of segmental speeds from mean race speed; CV expresses relative dispersion of pacing across splits, while pace range captures the difference between the fastest and slowest segments within a race. Together, these metrics can support a more consistent description of pacing behavior and may facilitate comparisons across race distances, performance levels, and athlete populations [78].

Positive pacing profiles are often interpreted as being consistent with fatigue-related decline [68,79], while negative pacing has been associated with markers of greater energy conservation and reduced physiological strain in longer events [70,80,81]. Greater pacing variability may be less efficient than steadier pacing, particularly on flat courses [22,56,66].

## 2. Materials and Methods

### 2.1. Study Design and Ethical Considerations

Ethical approval was not required because this narrative review was based exclusively on previously published literature. The conduct and reporting of the review were informed by published methodological guidance for narrative literature reviews [82,83] and by the Scale for the Assessment of Narrative Review Articles (SANRA) [84]. The review employed a structured but non-systematic search and narrative synthesis to identify and interpret studies examining pacing profiles, pacing behavior, and pacing variability in half-marathon and marathon running. Both male and female participants were considered. Findings were interpreted narratively according to the study design, sample size, race context, population characteristics, type of pacing data, and the comparability of split-segment structure across studies.

No formal methodological-quality appraisal, risk-of-bias assessment, certainty grading, or numerical weighting of the evidence was conducted. During the narrative synthesis, greater interpretive emphasis was placed on larger and more representative studies with directly relevant pacing outcomes and sufficiently detailed split data. Case analyses and studies with very small or highly selected samples were treated as illustrative rather than confirmatory evidence. Study-specific limitations relevant to interpretation are summarized in Table 1, Table 2 and Table 3.

### 2.2. Literature Search and Study Selection

**Figure 1:**
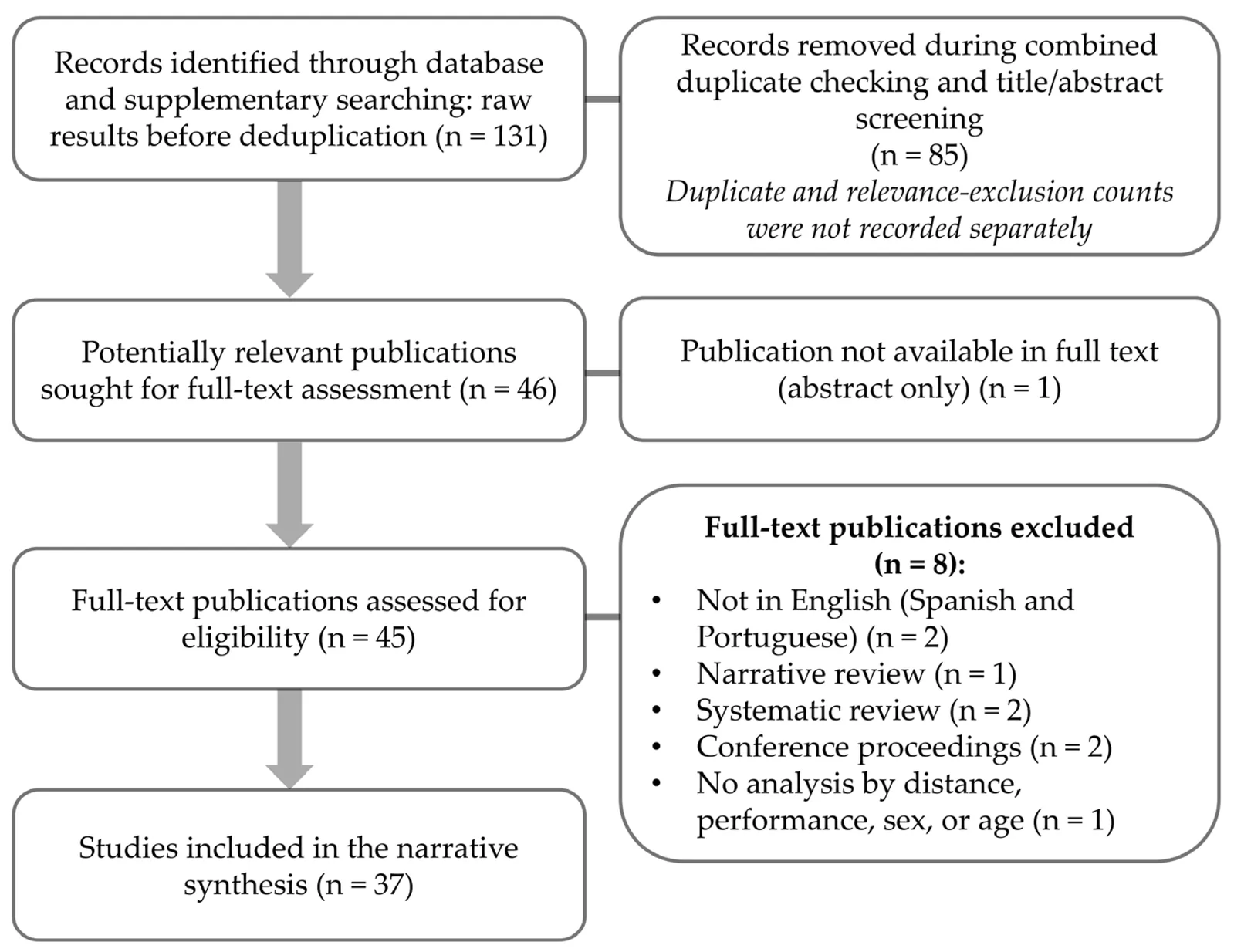
Identification and selection of studies included in the narrative synthesis. The 131 records represent the raw search yield before deduplication; duplicate removal and title/abstract exclusions were not documented separately.

The study-selection process is summarized in Figure 1. The initial search yield comprised raw records before deduplication. Because duplicate removal and title/abstract exclusions were not documented separately, they are presented as a combined stage, and the exact number of duplicates and unique records screened could not be reconstructed retrospectively.

PubMed, SPORTDiscus, Scopus, and Web of Science were searched from database inception to 31 March 2026, while Google Scholar was used as a supplementary source. Search terms were developed from the objectives of the review and terminology used in key publications on long-distance running and pacing. Candidate terms were organized into three conceptual blocks: race distance (“marathon”, “half-marathon”, “half marathon”, “long-distance running”, and “endurance running”), pacing (“pacing”, “pacing strategy”, “pacing behavior/behaviour”, “pacing profile”, “pacing pattern”, “running pace”, and “pacing variability”), and subgroup characteristics (“performance level”, “elite”, “recreational”, “sex”, “gender”, “age”, “age group”, and “master runner”). In the primary database searches, terms within each conceptual block were combined with OR, while the three conceptual blocks were combined with AND. The search strategy was pilot-tested and adapted to the search fields, syntax, and indexing structure of each database. Reference lists of relevant articles were also screened to identify additional studies where appropriate.

Records were initially screened by title and abstract for relevance to pacing behavior in half-marathon and/or marathon running. Potentially relevant publications were then assessed for eligibility, including full-text availability where applicable. The first author conducted the initial title and abstract screening, full-text assessment, and data charting. Cases in which eligibility or classification was uncertain were reviewed with a second author and resolved through discussion and consensus. Because this was a narrative review, screening was not conducted independently in duplicate, and no inter-rater reliability statistic was calculated.

No formal validated screening form or data-extraction instrument was used. However, information from eligible studies was charted in a structured spreadsheet containing bibliographic information, race distance, study design, sample size and participant characteristics, race setting, performance classification, sex and age variables, split structure, pacing metric, main pacing findings, and methodological considerations relevant to interpretation.

Studies were included if they examined pacing profiles, pacing behavior, pacing variability, split times, or race-speed changes in half-marathon and/or marathon runners, and if they reported findings according to race distance, performance level, sex, age, or a combination of these factors.

Studies were excluded if they did not include half-marathon or marathon running, did not report pacing-related outcomes, were not available in full text, were not published in English, or were meta-analyses, review articles, abstracts only, conference proceedings, or commentary papers.

When a study reported several race distances, only findings reported separately for the half-marathon and/or marathon were charted; findings combining eligible and ineligible distances without separate results were not used to support distance-specific conclusions. When multiple pacing metrics were reported, all relevant metrics were charted according to the construct represented, such as pacing-profile shape, segment-to-segment pace change, first-half versus second-half change, late-race decline, end spurt, or pacing variability. Metrics based on different definitions or split structures were not converted into a common effect measure or pooled quantitatively.

The study selection flow chart refers only to studies included in the main synthesis according to the predefined inclusion and exclusion criteria. Additional references addressing theoretical concepts, physiological mechanisms, environmental and course-related factors, contemporary technologies, or other contextual aspects of pacing were used to support the narrative discussion but were not counted among the included studies unless they met all eligibility criteria and directly reported pacing findings according to race distance, performance level, sex, or age.

The final narrative synthesis included 37 studies. Of these, one study examined pacing profiles in the half-marathon, 30 focused on marathon running, and six investigated pacing profiles across multiple race distances. Among the latter, five included both the half-marathon and marathon, whereas one compared 10 km, half-marathon, and marathon races and was retained only to contextualize distance-related pacing patterns relevant to the present review.

Across the included studies, pacing was quantified using heterogeneous split structures. The single study focused exclusively on half-marathon pacing used five race segments. Marathon studies most commonly used eight 5-km segments plus the final 2.195-km segment (nine race segments/splits), although several studies used half-race comparisons, 1-km segments, mile-based analyses, ten-segment models, or unequal race sections. Studies comparing different race distances generally used five race segments to facilitate between-distance comparisons, while one study used available race splits expressed as relative segment pace. These methodological differences were considered during the narrative interpretation of the findings.

## 3. Results and Discussion

### 3.1. Pacing Profiles in the Half-Marathon

#### 3.1.1. Pacing Profiles by Performance Level

Relatively few studies have examined pacing profiles in the half-marathon based on performance level [29,42]. Given this limited evidence base, the findings in this subsection should be interpreted as study-specific observations rather than as generalizable half-marathon pacing patterns. Only the study by Hanley (2015) [42] was included in Table 1, whereas the study by De Leeuw et al. (2018) [29], which also analyzed marathon and 10 km races, was included in Table 3. Among runners with a high-performance level, slightly negative and positive pacing profiles were observed, whereas Hanley reported a parabolic reverse J-shaped pacing pattern in elite championship runners [42]. Most elite runners completed the first 5 km relatively quickly, progressively slowed until around the 20 km mark, and then significantly accelerated during the final 1.1 km segment, whereas the fastest runners of both sexes largely maintained their pace between the fifth and 15th km [42]. In that study, running in a group was associated with a smaller decline in pace after the fifth kilometer, although this observation should be interpreted cautiously because inappropriate group pace may negatively affect pacing regulation [42]. However, Hanley’s study was observational and described pacing profiles using average segment speeds; none of the variables describing the magnitude of speed change were utilized, making it impossible to determine differences in pacing magnitude.

In recreational master runners, Piacentini et al. (2019) observed similar patterns: slower runners exhibited a pronounced decline in pace relative to their predicted finishing time, whereas faster or target-achieving runners maintained a more consistent pace [79]. Pacing profiles varied according to deviation from predicted finish time, while perceived exertion did not differ between groups. This study was not included in the tables or detailed analysis because it did not account for performance level, sex, or age.

One mass-participation study examining pacing in the half-marathon based on performance level also included the marathon, and its results are discussed in the section “Between-race differences in half-marathon and marathon pacing profiles” [33].

#### 3.1.2. Pacing Profiles by Sex and Age

According to available data, only one study compared pacing profiles between elite male and female half-marathon runners [43]. Although not included in Table 1 because it was published in conference proceedings, the findings showed that male runners exhibited a significant pace decline after the first 5 km. In contrast, female runners maintained a more even pacing profile. Both sexes experienced a decline as the race progressed, with a significant difference observed only between 10–15 km, where women ran relatively faster, followed by a marked slowdown after 15 km.

In Hanley (2015), male medalists showed slightly higher speeds in the middle segments compared to the start, while female runners demonstrated a slight decrease in speed until the end spurt, indicating a corresponding pacing profile [42]. However, since pacing was analyzed separately without direct sex comparison, the magnitude of these differences remains unclear.

Two other studies examining sex differences in pacing profiles among recreational half-marathon runners also included comparisons with marathon runners [69,103]. Notably, the only studies that examined pacing in half-marathons according to age also followed a similar approach, simultaneously analyzing marathon performance and comparing these two distances [104,105]. These comparisons are presented in Table 3, and the increasing number of participants across all age groups further indicates the need for more dedicated research on sex- and age-specific pacing patterns in the half-marathon.

Overall, studies on half-marathon pacing remain limited, particularly when subgroup factors such as performance level, sex, and age are considered, thereby hindering a comprehensive characterization of pacing behavior at this race distance. Therefore, current conclusions regarding half-marathon pacing should be interpreted as preliminary and less robust than those for marathon running. Although available evidence suggests that faster half-marathon runners may exhibit more stable pacing or controlled late-race acceleration, stronger subgroup-specific conclusions cannot yet be drawn.

### 3.2. Pacing Profiles in the Marathon

#### 3.2.1. Pacing Profiles by Performance Level

Several studies have examined pacing profiles according to performance levels in different marathon races (Table 2) [27,36,37,39,86,87,90]. Marathon running is generally characterized by progressive slowing, with higher-performing athletes showing smaller speed declines and lower pacing variability than slower runners [19,27,29,39,86,98]. This difference between performance groups appears to be independent of sex [27] and is further supported by evidence showing lower speed variability in athletes with higher aerobic capacity [75].

Elite, world-class runners are capable of negative pacing [87,94,95,101], and similar patterns have also been reported among higher-performance runners [41]. The prevalence of negative pacing decreases with performance level, being most common among top percentile runners and rare among slower athletes [41]. Women exhibit slightly higher rates than men, although these differences diminish at lower performance levels [41]. In line with this, negative split profiles may be associated with more favorable marathon performances, as they are associated with improved physiological efficiency, including reduced carbohydrate utilization, lower oxygen consumption, and decreased blood lactate concentrations [70,80,81].

In championship settings, men typically exhibit negative pacing, whereas women more often adopt conservative starts followed by end spurts [94]. However, in elite male marathoners, end spurts have been observed only in the slowest performance group [91], suggesting limited applicability of this strategy at the highest performance level. In marathon races in which world records were achieved, elite athletes generally maintained even pacing during the first half of the race and increased speed in the second half, frequently finishing with a final surge [87]. In contrast, slower runners are more likely to adopt an overly fast starting pace followed by progressive slowing [39,86], often culminating in “hitting the wall” during the later stages of the race [34,106,107]. These pacing patterns are consistent with, but do not directly demonstrate, previously described fatigue-related mechanisms [108,109], and coincide with greater pacing variability and poorer overall performance in slower runners [27,34,36,38,39,86,110].

In positive pacing, speed decline may reflect the interaction between perceived exertion and remaining distance [68,79]. Pacing regulation may be further affected by cognitive load [111]. Biomechanical changes, including reduced stride length and cadence [25,112] and increased ground contact time [112], may also contribute to pace reduction.

Early-race pacing has been strongly associated with finishing performance [85]. Furthermore, medalists in major championships typically maintain even pacing from approximately the 10-km mark onward, whereas lower-placing athletes tend to lose contact with the leading group around halfway [91]. Running in groups of similar performance level has been associated with more even pacing compared with running alone [42,91].

Overall, pacing variability decreases as performance level increases, while negative pacing and end-spurt behavior are more frequently observed among elite runners. In contrast, slower runners tend to exhibit greater fatigue-related declines. Although similar pacing profiles (e.g., reverse J-shaped) may be observed across performance levels, their underlying explanations may differ and should be interpreted inferentially. In elite athletes, such patterns may be consistent with more deliberate pace regulation, although the included observational studies cannot directly confirm intentional pacing strategy. Conversely, similar profiles in slower or recreational runners may be associated with less effective pace regulation or difficulty matching pace selection to physiological demands. Therefore, observed pacing profiles should be interpreted as indicators of pacing behavior rather than direct evidence of a deliberately planned strategy. This distinction is particularly relevant because large-scale observational studies provide the strongest support for the association between higher performance level and lower pacing variability, whereas small-sample, elite, and world-record analyses mainly offer contextual examples of pacing regulation under highly specific performance conditions.

#### 3.2.2. Pacing Profiles by Sex

In general, men consistently outperform women in running events, but the magnitude of the sex difference varies with race distance, increasing in sprint events and differing across endurance distances and performance levels [113,114,115]. As race participation has increased, average marathon pace has declined [3,8], especially among men [5], resulting in a reduced performance gap between male and female runners [5], particularly in longer races [6,41,116]. Additionally, women’s participation has increased more than men’s [4,5], while sex differences in performance decrease with age and lower ranking, alongside more even pacing in women [41].

The studies that analyzed sex-based pacing profiles among marathon runners are presented in Table 2. Previous research has reported significant sex differences in marathon pacing profiles among recreational runners [27,39,41]. These differences appear to be more pronounced at higher external temperatures [93] and less pronounced among faster runners [71]. However, such differences have not been consistently observed in elite runners [93].

Across performance levels, a positive pacing profile has generally been observed in both sexes [27,29,96], with exceptions reported for world record holders [94,100]. In recreational runners, women show a smaller pace decline [29,39,71,97,98,99].

One study found that women ran up to 4.5% faster than men in the final kilometers (from the 40th km to the finish line) [99], which may be partly related to men selecting an initial pace that was too fast relative to their ability; this may reflect overconfidence and contribute to greater pace decline [98]. However, even among elite female runners with relatively slower performances, a tendency to start at too fast a pace compared to individual abilities has been observed, possibly due to competitive pacing cues set by faster athletes [19].

When comparing male and female marathoners in the same performance groups, the pace decline in male runners is significantly more pronounced in the group with finishing times ranging from 2:15 h to 2:30 h, and in all performance groups from 3 to 6 h [27]. A significant difference between sexes was observed in the 25–30 km segment [75], characterized by a slight increase in speed among women and a continued decrease among men. However, negative [100] and even pacing strategies with acceleration at the end [92] were observed in male world record holders in the marathon, while female pacing profiles were less consistent [100] and negative with an end spurt among medal winners [91]. When the pacing profiles of the best female and male marathon performances were compared, they differed in speed time course, including trend and asymmetry, and in fractional use of critical speed [101]. In addition, women demonstrate even and negative pacing profiles across both world record and championship performances, often with pronounced end spurts in championship races, whereas men show a shift from even pacing in world record performances to more negative pacing in championship settings [94].

Women generally maintain more consistent pacing than men, who more often start too fast and slow down later. These differences are more pronounced in recreational runners and at higher temperatures but tend to diminish at the elite level.

#### 3.2.3. Pacing Profiles by Age

Since 1980, participation in marathons has increased among runners over 40 [5]. Although motivation for endurance running may vary with age [117], the highest participation remains in the 30–44 age group [4,5,7], which may be linked to physical motives, social influences, and available time [44]. The fastest runners are typically aged 30–44 [41]. Therefore, age-group comparisons in mass-participation races should be interpreted cautiously, as they may reflect cohort composition, participation patterns, and selection effects, in addition to biological aging.

Studies investigating pacing profiles across age groups in marathon runners are presented in Table 2. Previous research indicates some differences in pacing across age groups [27,38,97], although these effects are generally small or inconsistent [32,75], with largely similar pacing profiles observed across age categories [32,38,75].

Some studies suggest that older runners may maintain a more even pace than younger runners of similar performance level [27,38,97], particularly among lower-performing groups [38]. However, these differences are generally small, dependent on performance level, and may partly reflect greater experience and the positive selection of older finishers in mass-participation races [32,75,97]. Taken together, age-related differences in marathon pacing appear less consistent and less pronounced than those associated with performance level. They should therefore be interpreted with caution and in relation to performance status, training background, and running experience.

### 3.3. Between-Race Differences in Half-Marathon and Marathon Pacing Profiles

The importance of comparing pacing profiles across different long-distance races first emerged from a lack of clear explanations for the mechanisms underlying the significant decrease in running speed during the second half of an event (i.e., the positive split). Various authors have previously proposed physiological and psychological mechanisms as potential causes [36,51,71,73,118]. Given the influence of external conditions on pacing behavior [54,59,93], comparisons across races are most informative when environmental factors are similar.

In recent years, a new methodological approach has enabled direct comparisons of pacing profiles between half-marathon and marathon races held within the same event (Vienna and Ljubljana 2017), on the same day, at the same time, and on similar courses [33,69,103,104,105]. These studies are summarized in Table 3. Additionally, one study applied the same methodology to compare pacing profiles between marathon and 10-km races [102]. Across all races, runners generally showed progressive slowing in both sexes and across all age groups. As all of these studies are based on mass-participation events, their findings likely reflect predominantly recreational runners.

Most studies summarized in Table 3 compared half-marathon and marathon races held within the same event and under broadly similar race conditions, which supports a cautious interpretation of between-race pacing differences. However, direct comparability remains limited by differences in course design, participant composition, and split structure across studies and, in some cases, between race distances within the same study. One study used a multi-distance profiling approach across 10 km, half-marathon, and marathon races; therefore, its findings are used to contextualize distance-related pacing patterns rather than as direct statistical evidence of half-marathon–marathon differences.

Only one study analyzed sex differences in pacing profiles across 10 km, half-marathon, and marathon races, with a large sample [29]. Split times differed by race, but pacing profiles were not statistically compared across distances; analyses were descriptive, with age included as a predictor and results expressed relative to the median performance.

End-spurt findings should be interpreted cautiously, as an end spurt has been observed in marathon runners [105] and in half-marathons in Vienna races [33,69,104], whereas it was not consistently observed in Ljubljana races, possibly due to course characteristics [103,105]. End-spurt occurrence may therefore reflect race context, course characteristics, and performance level, and was more frequently observed in slower runners regardless of sex or race type [33].

Across the available multi-distance studies, lower pacing variability was generally observed in the half-marathon than in the marathon [69,103,104,105]. A similar pattern in pacing profiles was also observed in the 10 km race compared to the marathon [102]. Among men, lower pacing variation was observed in all performance groups of half-marathon runners compared to marathon runners, consistent with the previously described differences between race distances, whereas differences among women were of trivial practical significance [33]. Additionally, faster runners exhibited less pacing variation across both race types.

**Figure 2:**
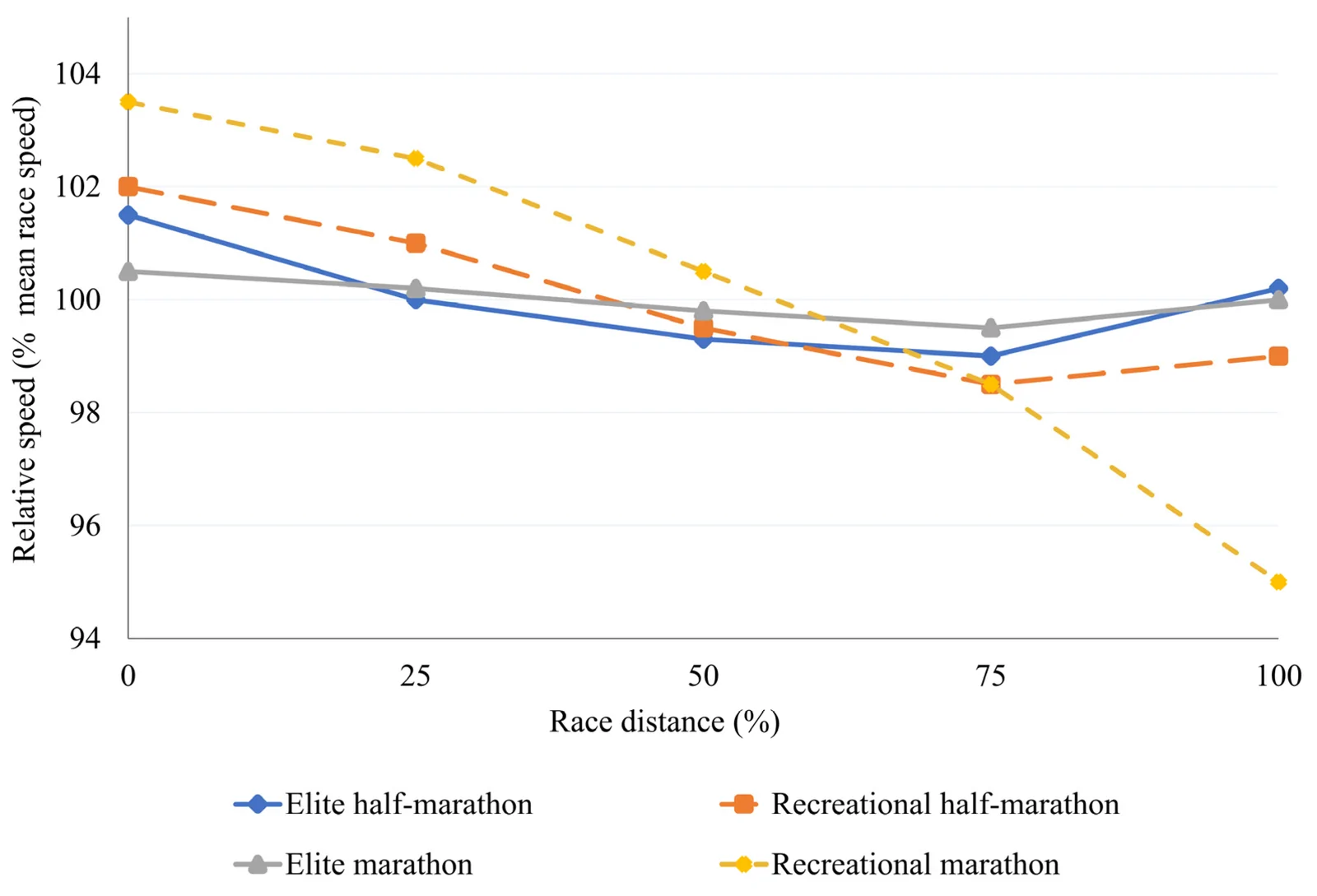
Schematic comparison of pacing profiles according to race distance and performance level. The figure illustrates commonly observed tendencies in the literature, with generally more even pacing in elite runners and in the half-marathon, and more pronounced late-race slowing in recreational marathon runners. The curves are illustrative and do not represent pooled quantitative estimates.

A schematic comparison of the main pacing tendencies according to race distance and performance level is presented in Figure 2.

These differences may reflect differences in pacing regulation mechanisms that integrate experience, environmental cues, and physiological constraints [22,49,68,71]. In this context, the longer duration of the marathon may be associated with greater physiological demands compared with the half-marathon [119]. These potential constraints may include energy/substrate availability and carbohydrate utilization [61,66,109], accumulated muscular strain [119,120], and rising perceived exertion as part of psychophysiological pacing regulation [22,49], which may contribute to more pronounced late-race slowing in recreational and lower-performing marathon runners [106,118]. This pacing behavior, particularly in recreational and lower-performing runners, may be interpreted as consistent with a process in which early pace regulation is guided by perceptual cues, whereas later-race slowing may increasingly reflect accumulating physiological constraints [22,49,121].

Because most included race-based pacing studies relied primarily on split times and segmental speeds and did not directly measure glycogen availability, hydration status, thermoregulatory strain, muscle damage, or neuromuscular fatigue, these mechanistic explanations should be regarded as inferential rather than as direct evidence. Consequently, more even or slightly negative pacing profiles have generally been associated with more favorable performance outcomes, although their effectiveness may depend on individual capacity and race conditions [81].

Environmental and course-related factors may further modify pacing behavior, particularly in the marathon. In this paragraph, findings from included race-based pacing studies are discussed together with broader contextual and mechanistic literature used to interpret possible environmental influences on pacing behavior. Course topography has been associated with pacing variation in elite marathon performances [53], while race course characteristics appear to have greater effects on pacing in slower time categories [89]. Climatic conditions, particularly higher ambient temperatures, may be associated with altered pacing, potentially through increased thermoregulatory strain, with stronger effects in longer races and in slower runners because of greater exposure time [55,56,59,93,122]. Humidity may further modify this response, especially under hot-humid conditions, although its independent association with pacing appears less consistent across marathon studies [54,58,59,122]. Hydration planning may also be relevant in recreational marathon runners, as slower and first-time runners have been reported to show less understanding of exercise-associated hyponatremia and potentially inappropriate fluid-intake plans [123]. Nevertheless, pacing profiles may remain relatively consistent across different editions of the same marathon, suggesting that environmental effects interact with, rather than replace, the influence of performance level, race distance, and athlete characteristics [88]. Collectively, these findings suggest that environmental conditions and course characteristics should be interpreted as contextual modifiers of pacing behavior rather than isolated determinants.

Although pacing is closely associated with endurance performance, its contribution to marathon prediction appears limited [124]. Half-marathon performance alone explains most of the variance in marathon results, while pacing variables add little, likely because pacing effects are already reflected in overall race time and shorter events may not fully capture fatigue-related declines relevant to marathon performance [124].

Cuk et al. (2019) [69] reported larger sex differences in pacing in the marathon than in the half-marathon. In contrast, Nikolaidis et al. (2019) [103] found no significant sex differences in half-marathon pacing, despite slightly higher absolute change in speed in women (4.11% vs. 4.09%). Similarly, no sex differences were observed in 10 km races [102]. Together, these findings suggest that sex differences in pacing may diminish with shorter race distances; however, the available evidence from half-marathon events remains limited, and larger samples, particularly of women, may reveal different patterns.

These observations suggest that multiple factors may contribute to sex differences in pacing variability. Psychological [98,125,126] and social factors [127] may partly account for these differences; however, their relative contribution cannot be determined from the included race-based pacing studies. Because sex differences in pacing appear to vary across race distances, physiological factors may also be relevant, particularly in longer races [128,129,130].

Potential physiological explanations for the observed sex-related pacing patterns include a higher proportion of type I muscle fibers [130,131], greater reliance on fat oxidation, and greater oxidative capacity in female runners [128,132,133,134,135], as well as faster oxygen extraction dynamics [129]. In contrast, male runners may rely more heavily on carbohydrates, partly because of a higher proportion of type II fibers [130,131], which may increase the risk of glycogen depletion during longer races (>~2 h) [108,109]. This may partly contribute to greater pacing variability, especially in marathons [69,103], and a higher prevalence of “hitting the wall” (~28% vs. ~17% in females) [106]. Additional potential advantages in female runners, such as better thermoregulation under rising race temperatures [136], may further influence pacing regulation in longer events, although overall performance may also be constrained by lower oxygen-carrying capacity and higher body fat percentage [113]. These physiological differences may provide plausible explanations for the observed sex-related pacing patterns, but they should be interpreted as inferential because most included race-based pacing studies did not directly measure these mechanisms.

In longer races and older age groups, the sex performance gap tends to decrease [6,116]. At the same time, female participation is higher at shorter distances and decreases as race distance increases [116], which may influence observed sex differences in pacing and performance.

Mass race data suggest that women may appear slower in the half-marathon than in the marathon, whereas the opposite trend is observed in men [105]. However, other studies report slower pacing in the half-marathon for both sexes [6], contrary to expectations based on race distance. This discrepancy may be related to participation structure, as increasing participant numbers are associated with lower average performance [3,8], and the higher female participation in half-marathons suggests a greater proportion of less experienced runners [4,6]. As a more accessible distance, the half-marathon may attract beginners with greater pacing variability [71,72], while marathon runners are more likely to follow structured training [137].

The data suggest that marathon participants tend to be older than half-marathon participants [6], particularly among men, whereas this pattern was not observed among women [105]. Peak performance age varies across studies [3,41,105], likely due to differences in sample composition and race context, which may also influence observed pacing patterns. Additionally, large-scale marathon data indicate that age-related patterns are influenced by participation structure and country of origin [7], which may affect the composition of age groups across events. Therefore, age groups in mass-participation road races should not be interpreted as purely biological categories. They may also reflect cohort composition, participation patterns, training history, running experience, and selection effects, because older finishers may represent a more experienced and positively selected subgroup of runners.

There was no difference in pace variability across age categories for female runners, while male runners younger than 30 years and older than 60 years showed greater variability compared to other age groups [104]. A similar pattern was observed in the study by Cuk et al. (2021) [102].

In the study by Cuk et al. (2019) [104], younger runners in the half-marathon exhibited a more pronounced end spurt compared to older age categories and marathon runners. In contrast, Nikolaidis et al. (2019) [105] did not identify consistent age-related differences in pacing and reported the presence of an end spurt in marathon races but not in most half-marathon age groups. This discrepancy may be partly explained by methodological differences between the studies, including differences in race context and sample size, as the study by Cuk, Nikolaidis, Markovic et al. (2019) included substantially more participants, particularly in the marathon [104]. Therefore, further research with larger samples is needed to clarify potential age-related differences, especially among female runners.

Although age-related findings are not entirely consistent, pacing differences across race distances appear to interact with age, with greater variability observed in longer events [104,105]. However, these patterns should not be attributed to biological aging alone but rather interpreted as reflecting the combined influence of race-distance demands [104,105], fatigue-related constraints [118], experience-related pacing regulation [49], age-related physiological changes [138,139,140,141,142,143], and differences in participation structure and sample composition [3,7,41,105].

Younger runners, particularly men under 30, tend to exhibit greater pacing variability, which may reflect overestimation of abilities, higher confidence, and limited experience [96], potentially impairing anticipatory effort control [49]. Environmental factors may further modulate these patterns, with temperature having a greater impact on middle-aged runners and humidity affecting the youngest and oldest athletes [59]. In contrast, greater pacing variability in older runners may be consistent with age-related declines in VO_2_max (~10% per decade) [138,139], reduced muscle mass and quality [140,141], and neuromuscular limitations affecting efficiency and recovery [142], which become more pronounced after ~50 years [141,143] and may be further exacerbated by reduced training [32]. However, in mass-participation races, these patterns may also be influenced by training history, race-selection effects, and the fact that older finishers may represent a positively selected subgroup of runners.

These age-related patterns appear to be more pronounced in the marathon than in shorter distances [29]. Overall, both younger and older runners may show greater pacing variability, particularly in longer events. However, age should be interpreted as a secondary contextual factor in pacing behavior, with observed differences likely reflecting a combination of biological aging, cohort effects, participation structure, running experience, and selection effects.

### 3.4. Contemporary Technologies and Modern Marathon Pacing

Recent developments in marathon running may influence the interpretation of modern pacing profiles. This subsection is presented as contextual discussion rather than as a synthesis of factors that were consistently examined as primary variables in the included half-marathon and marathon pacing studies.

Advanced footwear technology, including carbon plates and highly responsive foams, has been associated with faster road-race performances and changes in running economy and biomechanics [9,10]. Recent evidence from World Marathon Majors suggests that these technologies may also be related to pacing behavior, although effects may differ by sex: in men, advanced footwear technology appears to be associated mainly with higher running velocities without substantially altering pacing patterns, whereas in women it may be associated with faster initial speeds and a steeper late-race decline [144]. In addition, pack running, professional pacing support, and optimized race organization may facilitate more stable pacing in elite or record-attempt contexts [91,92]. Controlled pacing aids, including light- or vehicle-assisted pacing, have been used in non-standard record-attempt settings, but their direct relevance to official marathon pacing remains less established. Therefore, contemporary marathon pacing profiles should be interpreted within the context of footwear technology, pacing assistance, pack dynamics, and race organization, rather than solely as reflections of athletes’ physiological capacity or planned pacing strategy.

### 3.5. Synthesis of Pacing Determinants and Contextual Modifiers

**Figure 3:**
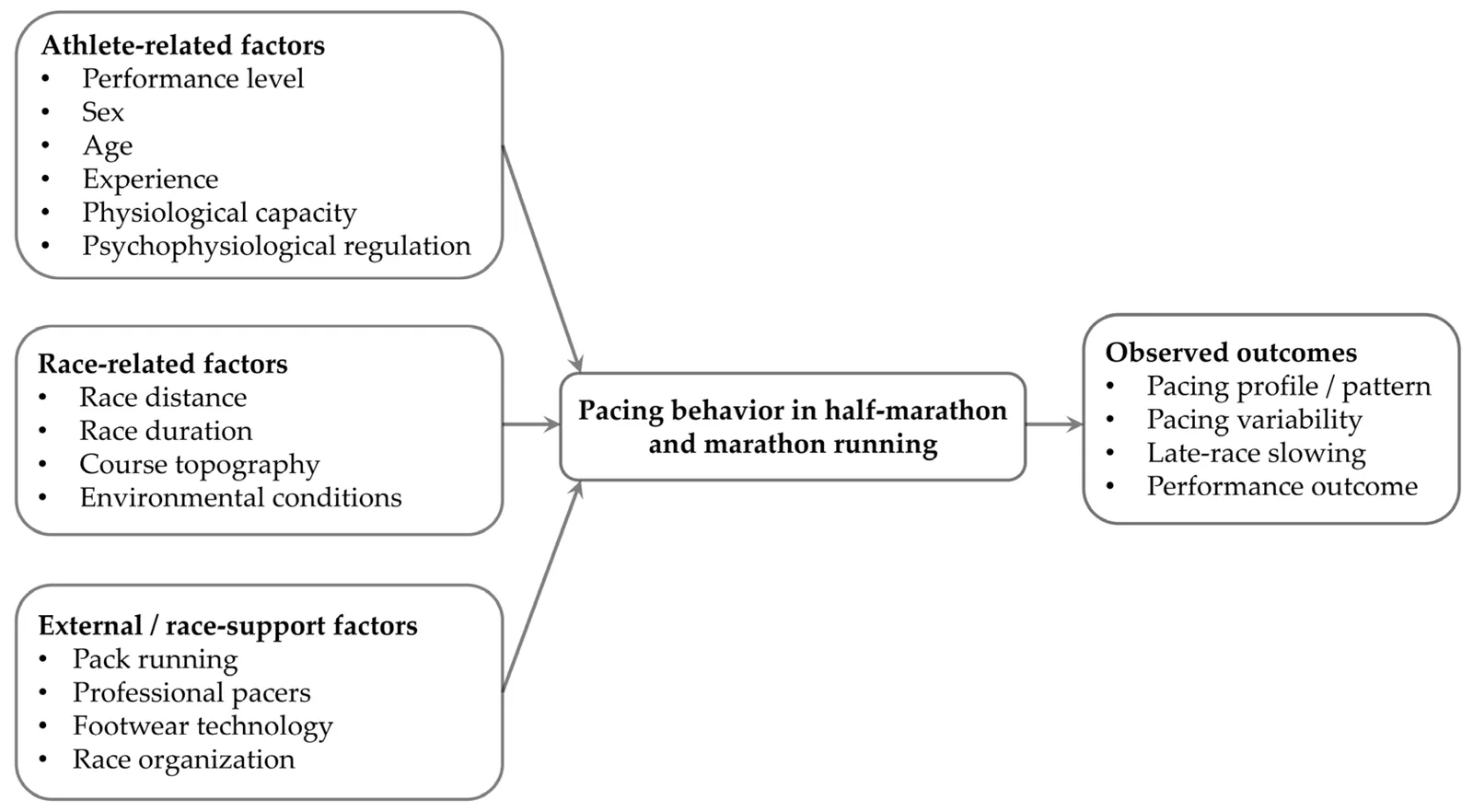
Conceptual model of determinants and contextual modifiers of pacing behavior in half-marathon and marathon running. Pacing behavior is influenced by athlete-related factors, race-related factors, and external or race-support factors, which interact to shape pacing profiles, pacing variability, late-race slowing, and performance outcomes.

Based on the evidence discussed above, the main determinants and contextual modifiers of pacing behavior in half-marathon and marathon running are summarized in Figure 3.

### 3.6. Practical Implications for Coaching Practice and Recreational Runners

To enhance practical applicability, the main findings were translated into practical reference points and summarized in a concise coaching guide. Because most available evidence is cross-sectional and observational, the guide should be interpreted as a synthesis of observed associations rather than as a causal or normative model. Performance level and race distance appear to provide the most useful starting points for interpreting pacing behavior, while sex and age should be considered as secondary contextual factors (Table 4).

For recreational runners, these findings suggest the importance of realistic pace selection, particularly in the early race stages. This is especially relevant in the marathon, where overly ambitious starting speeds may contribute to greater late-race slowing, higher pacing variability, and less favorable race outcomes.

Overall, pacing behavior may be interpreted primarily according to performance level and race distance, as these factors show the clearest and most consistent patterns of pacing variability and late-race slowing. Sex- and age-related patterns may help refine recommendations, but they should be applied cautiously and in combination with individual race history and training background.

### 3.7. Limitations

This narrative review has several limitations. First, most available evidence is derived from observational, largely cross-sectional studies, which precludes causal interpretation of the reported associations, including those summarized in the practical coaching guide. Second, most studies used split-based analyses, but differences in how race segments were defined, together with differences in pacing metrics, race settings, participant characteristics, performance classifications, sample size, study design, and analytical approaches, complicated direct comparisons across studies. This heterogeneity was considered narratively during interpretation; however, no formal methodological-quality appraisal or risk-of-bias assessment was performed, which limits the strength of comparative claims across studies and subgroups. Although greater interpretive emphasis was placed on larger and more directly relevant observational studies, findings from case analyses and very small or selected samples should be regarded as illustrative rather than confirmatory. Third, the narrative design and absence of a formal quantitative synthesis limited the ability to provide precise estimates of pacing characteristics or subgroup-specific differences across performance level, sex, and age. In addition, the absence of a separate duplicate-removal log limited the retrospective auditability of the study-selection process.

Finally, the literature on half-marathon pacing remains considerably smaller than that on marathon pacing, further constraining comparisons across race distances. Therefore, the proposed coaching guide should be interpreted as an evidence-informed synthesis of observed patterns rather than as a prescriptive, causal, or normative model.

## 4. Conclusions

This review synthesizes evidence on pacing in half-marathon and marathon running and highlights how pacing behavior varies by race distance, performance level, sex, and age. Collectively, the available data indicate that pacing is generally more even in the half-marathon and more positive in the marathon, with performance level emerging as the factor most consistently associated with pacing behavior. Given the heterogeneity of the included evidence, conclusions regarding marathon pacing are more consistent because they are supported by a larger number of studies, whereas findings for the half-marathon, sex-specific patterns, and age-related differences should be considered more preliminary.

Faster runners tend to maintain a more stable pace, often characterized by even or slightly negative pacing profiles. In contrast, slower runners show greater variability and a more pronounced decline in speed. Sex differences are more evident in the marathon, where women generally show lower pacing variability than men, while age-related differences appear relatively small and inconsistent.

In mass-participation races, observed pacing profiles do not necessarily reflect a planned pacing strategy, particularly in recreational runners, where pacing behavior may be associated with fatigue-related changes and less accurate pace selection.

Participation structures may influence observed sex differences in pacing and could differ in studies with larger female samples across both race distances, particularly in marathon events.

Several questions remain unresolved. Evidence on half-marathon pacing remains limited, particularly regarding the combined influence of performance level, sex, and age, as well as the structure of female participation. In addition, the relative contributions of pacing decisions, fatigue-related physiological constraints, environmental conditions, and race-support factors to pacing variability among recreational runners remain unclear. Future studies using larger and more representative samples, particularly of female runners, higher-resolution data, and standardized pacing metrics would help clarify these issues. The practical reference points proposed in this review should therefore be viewed as tentative, evidence-informed aids for interpreting pacing behavior according to performance level, race distance, sex, and age, rather than as prescriptive recommendations.

## Figures and Tables

**Table 1:** Study examining pacing in the half-marathon by performance level.

Study	Main Factor	Sample/Context	Pacing Data/Design	Key Pacing Finding	Key Methodological Considerations
Hanley, 2015 [42]	Performance level	*N* = 838 (347 females); elite runners; six IAAF World Half-Marathon Championships 2007–2014.	Five race segments; mean speed per segment. Observational study using publicly available championship data.	Elite half-marathon runners displayed a reverse J-shaped pacing profile, with a fast start, gradual slowing until ~20 km, and a final end spurt. Slower runners showed greater speed decline after the first 5 km.	Elite championship sample; limited generalizability to recreational runners; pacing magnitude/variability not quantified beyond segmental mean speeds.

Note: Key methodological considerations summarize the main interpretative limitations of the study and should not be interpreted as a formal risk-of-bias assessment. *N* = number of participants; IAAF = International Association of Athletics Federations.

**Table 2:** Summary of key findings from studies investigating marathon pacing according to performance level, sex, and age. Studies are grouped according to the main factor investigated, and only the main pacing-related findings relevant to this review are presented.

Study	Main Factor	Sample/Context	Pacing Data/Design	Key Pacing Finding	Key Methodological Considerations
Thuany et al., 2025 [85]	Performance level	Not reported; New York City; 2011–2019; grouped by performance (1st–3rd, 4th–10th, >10th position).	Mile splits from mile 4 to 26; first-to-last mile change.	Higher-placing men showed lower pacing variability, whereas lower-placing men showed greater late-race decline. Women showed higher variability, and early pace was more strongly associated with final-mile pace.	Participant-level characteristics and sample size not reported; early race miles unavailable.
Muñoz-Pérez et al., 2020 [36]	Performance level	*N* = 2295 (637 females); men ≤ 3 h; women ≤ 3:30 h; Berlin, 2017; flat course.	Ten split times; positive/even/negative pacing.	Male runners showed relatively similar pacing profiles across performance levels, whereas faster female runners showed lower pace variation.	Restricted to faster finishers; limited generalizability to slower recreational runners.
Pycke & Billat, 2022 [37]	Performance level	*N* = 3; selected world-class marathon performances.	1-km splits; speed asymmetry and race-speed oscillations.	World-class runners displayed race-speed oscillations, with a fast start and prolonged running slightly below mean race speed.	Very small selected world-class sample; illustrative case analysis.
Santos-Lozano et al., 2014 [86]	Performance level	*N* = 190,228 (69,316 females); New York City; 2006–2011.	Nine split speeds; CV-based pacing variability.	All runners showed positive pacing, but faster runners had lower speed variability than slower runners.	NYC-specific event context; performance-level comparisons may reflect unmeasured participant differences.
Díaz et al., 2018 [35]	Performance level	*N* = 15; world records for men between 1967 and 1988 (classic athletes) and between 1988 and 2018 (contemporaneous athletes).	Nine race splits; relative segment speed.	World-record performances generally showed a faster first half followed by progressive slowing, with earlier records showing greater late-race speed decline than more recent records.	Male world-record performances only; historical context limits generalizability.
García-Manso et al., 2021 [87]	Performance level	Total = 235 races; Frankfurt subset: n = 110 top-10 male finishers, 2008–2018; additional sub-2:04:00, world-record, and record-attempt performances.	Nine race splits; tactical pattern grouping.	World-record performances generally showed controlled first-half pacing followed by increased speed in the second half and a final surge.	Selected elite male and record-attempt performances; limited generalizability to mass-participation runners.
Renfree & St Clair Gibson, 2013 [19]	Performance level	*N* = 60; women’s IAAF World Championship Marathon, 2009.	Intermediate and final times converted to mean speeds; segmental relative speed expressed as % of PB speed; performance groups based on finishing-placement quartiles.	The highest-placing group ran more conservatively early and relatively faster after 35 km, whereas lower-placing groups showed greater speed reductions.	Elite female championship context; tactical race setting may influence pacing.
Oficial-Casado et al., 2022 [88]	Performance level	*N* = 282,808 (no personal data); Chicago, London, and Tokyo marathons, 2017–2019.	Split speeds expressed relative to mean race speed; 30-min performance categories from 2:30 to <6:00 h.	Faster runners showed lower speed variability, and pacing profiles were highly consistent across marathon editions.	No personal data; event-specific course and weather effects difficult to separate.
Angus, 2014 [60]	Performance level	*N* = 2 males; two world-record marathon runs: run 1 (2:03:59, Berlin, 2008) and run 2 (2:03:38, Berlin, 2011).	1-km speed changes; course gradient and wind considered.	Headwind, but not gradient, was associated with speed variability; the two world-record performances showed distinct non-even patterns: Runner 1 was U-shaped, whereas Runner 2 was sinusoidal.	Two selected world-record cases; model-dependent interpretation.
Oficial-Casado et al., 2020 [89]	Performance level	*N* = 91,493 (no personal data); Valencia, Chicago, London, and Tokyo, 2018.	Nine split speeds expressed relative to mean race speed; six performance groups from sub-2:30 to sub-5:00 h.	Faster runners showed lower speed variability. Race-course characteristics were associated with pacing differences, particularly in slower time categories.	No personal data; course-related effects may be confounded by event-specific characteristics.
Myrkos et al., 2020 [90]	Performance level	*N* = 15; Athens; 2018; moderate-level group (finishing times < 240 min, n = 8), and a low-level group (finishing times > 240 min, n = 7).	Pre-race laboratory incremental test; race velocities at 10 km, 21.1 km, 30 km, and finish.	Low-level runners showed greater pace variability and steeper decline, particularly between 21.1 and 30 km; medium-level runners were less affected by hilly terrain.	Very small sample; sex data not reported; rolling-hill course limits comparability.
Hanley, 2016 [91]	Performance level, sex	*N* = 1222 (549 females); eight IAAF World Championships 2001–2015 and the Olympic Games 2012.	Nine race splits.	Medalists maintained more even pacing from ~10 km onward; women showed less second-half slowing, more frequent negative splits, and a final 2.2-km acceleration among medalists.	Elite championship context; limited generalizability to recreational runners.
Muñoz-Pérez et al., 2023 [92]	Performance level, sex	*N* = 279 (130 females); Berlin, 2008–2018, including five WRs plus two previous WRs and the second-fastest performance on the same course.	Ten race segments, including half-marathon and final 2.2-km segments; pacing and packing analysis.	Even pacing was associated with faster male performances. Women showed less second-half speed decline than men, especially after 35 km.	Selected elite performances on one course; pack-running context may influence pacing.
Trubee et al., 2014 [93]	Performance level, sex	*N* = 32,121 (12,068 females); finishing time < 5 h; Chicago; 2007 and 2009; flat course.	Last-12.195-km speed relative to first 30 km; sex and temperature effects assessed by regression.	Non-elite women showed more even pacing than non-elite men, and this sex difference increased at warmer race temperatures. No sex difference was observed among elite runners.	Coarse two-section pacing metric; two race years from one event.
Casado et al., 2024 [94]	Performance level, sex	12 men’s and 8 women’s WRs, and 14 men’s and 14 women’s World Championship/Olympic gold medal performances.	5-km split-speed changes.	Men’s world-record performances showed faster early speeds and more even pacing than Championship races. Women showed even-to-negative pacing in both contexts.	Small subgroup sizes limit sex- and context-specific comparisons.
Erdmann & Lipinska, 2013 [95]	Performance level, sex	*N* = 400 (200 females); top-50 male and female finishers from four international elite marathon events, 2001–2004; Gebrselassie’s 2007–2009 marathons analyzed separately.	5-km split times, half-marathon times, and final 2.195-km segment; ranking-group analysis; 1-km split data for Gebrselassie’s races.	Men’s velocity curves were ascending in the top 3, relatively even in the top 10, and descending in the top 50, whereas women generally showed progressive slowing. Gebrselassie’s Berlin performances showed ascending velocity trends.	Top-finishers only; selected elite events limit generalizability.
Nikolaidis & Knechtle, 2017 [38]	Performance level, age	*N* = 298,082 (117,595 females); New York City; 2006–2016; 5-year age groups from 18–84 years.	Nine race splits.	Older runners showed slightly more even pacing than younger runners of similar performance level, particularly among slower runners.	Single-event dataset; age-group findings may reflect differences in who finishes the marathon.
Nikolaidis & Knechtle, 2019 [32]	Performance level, age	*N* = 298,082 (117,595 females); New York City; 2006–2016.	Nine race splits.	The slowest runners showed the greatest early pace decline and largest end spurt, whereas the fastest runners showed the most even pacing.	Single-event dataset; age-group findings should be interpreted in relation to performance level.
Latorre-Román et al., 2025 [34]	Performance level, sex, age	*N* = 146,108 (23,565 females); Valencia; 2014–2023; 11 performance levels.	Nine race splits; relative speed, split-to-split speed change, CV, pacing profile (% change 1st vs. 2nd half), hitting the wall, and end spurt.	Women and faster runners showed more even pacing and lower CV; however, women had a higher prevalence of hitting the wall, whereas faster finishers had a lower prevalence. Even pacing decreased with age, and marathon time correlated with CV.	Single-event dataset; lower female representation.
Breen et al., 2018 [96]	Performance level, sex, age	*N* = 31,762 (11,743 females); New York City; 2015.	Positive range, negative range, pace range, and end spurt.	Pace range increased as performance level declined. Women showed smaller pace range than men except in the fastest group; age-related patterns were inconsistent.	Masters-only sample; age-related findings may be influenced by performance level.
March et al., 2011 [97]	Performance level, sex, age	*N* = 319 (134 females); Midwestern United States races; 2005–2007.	Mean velocity ratio between the final 9.7 km and first 32.5 km; 26 × 1.6-km loop course; stepwise multiple regression.	More even pacing was associated with faster finish times. Women, older runners, and faster runners showed more even pacing after adjustment.	Coarse two-section pacing metric; smaller regional sample from a loop-course setting.
Nikolaidis & Knechtle, 2018b [73]	Performance level, sex, age	*N* = 156 (26 females); Athens Classic Marathon 2017; elevation changes: 7–244 m.	Laboratory measures, split times from 5 km to finish, CV, and pace range; cross-sectional design.	Participants generally showed positive pacing with an end spurt. Women, older runners, and faster recreational runners showed more even pacing.	Small sample; low female representation; hilly course.
Kais et al., 2019 [27]	Performance level, sex, age	*N* = 116,670 (46,856 females); veterans = 55,059, non-veterans = 61,611; London, Chicago, and New York; finishing time ≤ 6 h; elevation difference: 8–55 m.	First-half vs. second-half pacing difference; regression adjusted for sex, age, and time group.	Faster finishers and women showed more even pacing. Veteran runners showed only slightly more even pacing after adjustment.	Limited split resolution (first-half vs. second-half comparison only); veteran/non-veteran grouping may oversimplify age-related effects.
Hubble & Zhao, 2016 [98]	Sex	*N* = 6676 (2521 females); Chevron Houston Marathon 2013.	Predicted finish time, second-half slowdown, and split pace deviation.	Men showed greater second-half slowing than women and larger discrepancies between predicted and actual finish times.	Single-event sample; predicted finish time may reflect inaccurate self-assessment.
Hernando et al., 2020 [99]	Sex	*N* = 88 (14 females); recreational runners; Valencia; 2016; flat course.	Accelerometer-based measures plus segmental and absolute speed change across nine race segments; repeated-measures design with online survey.	Women maintained more even pacing up to 40 km and showed a faster end spurt than men.	Small sample; low female representation; single-race context.
Díaz, et al., 2019 [100]	Sex	*N* = 18 (8 females); world record holders from 1998 to 2018.	Nine race splits; relative segment speed and CV.	Male world-record performances showed negative pacing, whereas female performances showed less consistent pacing with an end spurt.	Very small world-record sample; selected performances only.
Deaner et al., 2015 [71]	Sex	*N* = 91,929 (38,130 females); 14 marathons held in the United States, 2011.	Second-half pace change; maintaining pace defined as <10% slowing and marked slowing as ≥30%; sex-adjusted analysis using women’s chip times divided by 1.12.	Women had lower odds of marked slowing than men after adjustment for age and ability; this sex difference was smaller among faster runners and persisted after controlling for running experience.	Limited split resolution (first-half vs. second-half comparison only); sex-adjustment model may affect interpretation.
Billat, et al., 2020 [101]	Sex	*N* = 3 (2 females); world’s best results; Eliud Kipchoge (Berlin 2018 and London 2019), Brigid Kosgei (Chicago 2019) and Paula Radcliffe (London 2003).	Speed relative to critical speed; CV, asymmetry, and global race-speed trend.	Kipchoge showed negative-split characteristics, whereas the female runners displayed comparatively constant pacing.	Very small selected sample; illustrative case analysis.
Nikolaidis & Knechtle, 2018a [75]	Sex, age	*N* = 48,565 (20,283 females); New York City; 2015.	Nine race splits; multivariate regression.	Speed declined across the race in both sexes and all age groups, with greater decline in men, particularly around 25–30 km; age-group differences were trivial.	Single-event dataset; age-group interpretation may be influenced by participation structure.
Cuk et al., 2021 [102]	Sex, age	*N* = 8828 (2048 females); Oslo; 2015–2018.	Second-half vs. first-half speed variation.	Marathoners showed positive pacing. Women slowed less than men, and pacing changes were more pronounced in the youngest and oldest runners.	Limited split resolution (first-half vs. second-half comparison only); single-event dataset.

Note: Most studies were observational analyses based on publicly available race data unless otherwise stated in the pacing data/design column. Key methodological considerations indicate the main interpretative limitations relevant to this review and do not represent a formal risk-of-bias assessment. *N* = number of participants; CV = coefficient of variation; PB = personal best; WR = world record.

**Table 3:** Summary of studies examining pacing profiles in half-marathon and marathon running across different race distances, performance levels, sex, and age groups.

Study	Main Factor	Sample and Event Context	Pacing Data/Design	Key Distance-Related Pacing Finding	Key Methodological Considerations
Ristanović et al., 2023 [33]	Performance level	Total *N* = 208,760, marathon *N* = 75,492, half-marathon *N* = 133,268; Vienna, 2006–2018.	Five segment speeds; speed change and ACS.	Faster runners showed more consistent pacing in both races. Half-marathon runners showed more even pacing than marathon runners, although differences were small, particularly among women.	Long study period; edition-to-edition differences may affect performance-group comparisons.
De Leeuw et al., 2018 [29]	Performance level, sex	Total *N* = 98,563, marathon *N* = 79,638, half-marathon *N* = 18,925; Boston Athletic Association races, 2015–2017.	Half-marathon 8/16-km splits and marathon 5-km plus halfway splits; relative segment pace; multi-distance clustering and subgroup discovery.	Pacing profiles varied across distances: proficient half-marathon runners showed slight negative or positive pacing, whereas marathon runners generally showed positive pacing; lower speed decline was characteristic of faster runners.	Different split structures across distances; no direct statistical half-marathon–marathon comparison; race-course information not reported.
Cuk et al., 2019 [69]	Sex	Total *N* = 17,525, marathon *N* = 6085, half-marathon *N* = 11,440; Vienna, 2017.	Five segment speeds; speed change and ACS.	Half-marathon pacing was more even than marathon pacing. Women showed more even pacing than men, but sex differences were smaller in the half-marathon.	Single event/year; sex-specific findings may reflect event-specific participation structure.
Nikolaidis et al. 2019 [103]	Sex	Total *N* = 9137, marathon *N* = 1853, half-marathon *N* = 7258; Ljubljana, 2017.	Five segment speeds; speed change and ACS.	Half-marathon runners showed more even positive pacing without an end spurt, whereas marathon runners showed greater pacing variability with an end spurt; ACS was similar between sexes in the half-marathon but lower in women in the marathon.	Smaller marathon sample; findings limited to one race context.
Cuk et al., 2019 [104]	Age	Total *N* = 17,525, marathon *N* = 6081, half-marathon *N* = 11,384; Vienna, 2017.	Five segment speeds; pace range and end spurt.	Marathon pacing was more variable than half-marathon pacing across age groups; variability was higher in younger and older men, and younger half-marathon runners showed the fastest end spurt.	Single event/year; age-group findings may reflect participation structure.
Nikolaidis et al., 2019 [105]	Age	Total *N* = 9137, marathon *N* = 1853, half-marathon *N* = 7258; Ljubljana, 2017.	Five segment speeds; pace range and end spurt.	All age groups showed positive pacing. Pacing was generally more even in the half-marathon, whereas an end spurt was observed mainly in the marathon.	Smaller marathon sample; age-group findings may reflect participation structure.

Note: Unless otherwise stated, studies were observational analyses based on publicly available race data, and only the main pacing-related findings relevant to the comparison between half-marathon and marathon running are presented. In the Vienna studies, the race course was flat, with an elevation difference of 50 m; in the Ljubljana studies, the race course was flat, with an elevation difference of 29 m. In these studies, the half-marathon and marathon races were held within the same event, and the half-marathon course was entirely or largely contained within the marathon course. Key methodological considerations indicate the main interpretative limitations relevant to this review and do not represent a formal risk-of-bias assessment. *N* = number of participants; ACS = absolute change in speed.

**Table 4:** Practical reference points for coaching based on observed associations between pacing behavior, performance level, race distance, sex, and age.

Factor	Observed Pacing Tendency	Potential Practical Consideration
Faster runners	Lower pacing variability and smaller late-race decline are more commonly observed.	Even or slightly progressive pacing targets may be considered, while avoiding an excessively fast start.
Slower runners	Greater positive pacing, higher variability, and larger late-race decline are more commonly observed.	Conservative early pacing may be considered; “banking time” may be approached with caution.
Half-marathon	Pacing is generally more even than in the marathon.	A controlled target pace from the start may be considered, with less emphasis on aggressive early conservation than in the marathon.
Marathon	Positive pacing and late-race slowing are more common, especially in recreational runners.	Greater first-half pace control may be considered, particularly before the 25–35 km section.
Sex	Women generally show more even pacing than men, especially in recreational marathon samples.	Sex may help contextualize pacing interpretation but should not be considered independently of performance level.
Age	Age-related differences are small and inconsistent.	Age alone may be insufficient to guide pacing strategy; training history, experience, and previous race data may also be considered.

## Data Availability

No new data were created or analyzed in this study. Data sharing does not apply to this article.

## Abbreviations

The following abbreviations are used in this manuscript:

VO_2_max	maximum oxygen uptake
ACS	absolute change in speed
CV	coefficient of variation
*N*	number of participants
IAAF	International Association of Athletics Federations
PB	personal best
WR	world record
